# Lithium-induced neuroprotection in bipolar disorder: Translational insights into cytoskeletal and proteomic mechanisms

**DOI:** 10.1016/j.ibneur.2025.10.003

**Published:** 2025-10-09

**Authors:** Muthusamy Thangavel, T. Sterlin Raj, Sasikala Chinnappan, Muralidharan Anbalagan, Narasingam Arunagirinathan, Edal Kuvin, Pattabi Sasikumar, Chitraabaanu Paranjothy, Ravishankar Ram Mani

**Affiliations:** a Cellular and Molecular Biochemistry, Research and Development Wing, Sree Balaji Medical College and Hospital, Bharath Institute of Higher Education and Research (BIHER), Chromepet, Chennai, Tamil Nadu 600044, India; b Department of Pharmaceutical Biology, Faculty of Pharmaceutical Sciences, UCSI University, Cheras, Kuala Lumpur 56000, Malaysia; c Department of Structural & Cellular Biology, Tulane University School of Medicine, New Orleans, LA, USA; d Central Research Laboratory, Meenakshi Academy of Higher Research and Education (Deemed to be University), Chennai, India; e Department of Psychiatric Nursing, Bethel College of Nursing, Ranchi, Jharkhand State, India; f Department of General Surgery, Sree Balaji Medical College and Hospital, Bharath Institute of Higher Education and Research (BIHER), Chromepet, Chennai, Tamil Nadu 600044, India; g Klinik Chong (Medicruz Sdn. Bhd), No:55, 2nd Floor, Bangunan Eng Choon, Jalan Ampang, Kuala Lumpur 50450, Malaysia

**Keywords:** Lithium, Bipolar Disorder, Mood episodes, Depression, Neurodegeneration, Cytoskeletal Proteins

## Abstract

The pharmacotherapy of lithium plays mainstay in the modulation of bipolar disorder (BD) and controls other neuropsychiatric conditions such as prophylactic and mood episodes. However, its neurotrophic properties embrassing hypothetically converged synaptic regulation to BD remains elusive. Several studies intricate evidence of lithium targetting ‘microtubular associated protein-2 (MAP2)’ to endure neuroprotection. Synthesis of multiple single nucleiotide gene polymorphisms of *Snap-25* gene is considered a milestone in MAP2 alteration. In fact, upregulated NAA (N-acetyl-aspartate), oxidative stess, and neurotrophins can cause neuronal vulnerability. Proteomic data from animal models and human samples exhibited evidence of cytoskeletal proteins associated lithium-dependent neural plasticity. Recent findings emphasized significance on lithium controlling Akt and GSK3 inhibition as a crucial mechanism to promote neuroprotection. As a therapeutic regimen to BD, lithium targets gene expression regulation and post-translational modification pathways. Therefore this narrative review intent to summarise a comprehensive understanding of neurotrophic effects of lithium via diverse signaling pathways. Considering these steps we propose that the therapeutic effect of lithium can particularly modulate any one subtype of BD during the onset of sensitization. This scheme can further assist whether molecular pathways involving cytoskeletal regulation are amendable to treat BD. The review will also highlight stratified chemical pathways by which lithium action as an effective mood-stabilizing agent to exert its association with neural plasticity.

## Highlights

● Lithium as an effective drug for Bipolar disorder (BD).
● Multidrug approach of Lithium to neurodegenerative problems.
● Role of Lithium in neuropsychiatric treatments.
● Lithium enrich neuroprotection via cytoskeleton regulation.

## Graphical Abstract

**Figure:**
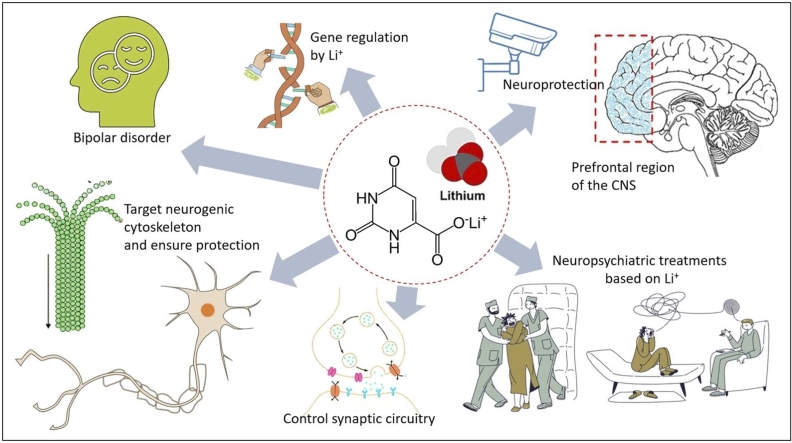

## 1 Introduction

Bipolar disorder (BD) is one of the devasting mood disorders influenced by manic-depressive neuropsychiatric condition affecting ∼2 % of the global population (Kessler et al., 2005). Despite the fact that neurotransmitter systems manages both visceral and behavioural symptoms of mood, BD may be a condition of disrupted synapses and circuits, particularly in the region of prefrontal cortex of the brain. Several pathophysiological observations anticipated BD as a cause of cellular injury and/ or due to dysregulated cellular mechanisms. Lithium (lightest of all metals) explicit intense manifestation to control BD progression via specific mechanisms underlying cellular circuitry remains a hot topic of study (Tondo and Baldessarini, 2009, Muller-Oerlinghausen and Lewitzka, 2010). As a monovalent cation, lithium weighs half the density of water and is considered to be a standard treatment regimen for BD over the past 60 years. It works via inhibiting inositol polyphosphate1-phosphatase (IPP), and inositol monophosphatase-1 (IMPA1) resulting accumulation of high quantity inositol 1-phosphates in the neuronal cavity. This inhibition eventually reduces myo-inositol assembly and lowers the level of cellular inositol triphosphate thus act as a key regulatory factor to the secondary messenger synthesized by the membrane bound phosphor-inositides (Williams and Harwood, 2000, Beaulieu et al., 2009). In this way lithium influence the glycogen synthase kinase-3 (Gsk3), and/or Akt (protein kinase B) signaling mechanisms (Williams and Harwood, 2000, Baldessarini and Tondo, 2000) to affect several neurotropic factors (Gould et al., 2004, Wada, 2009) such as the cytoskeletal framework, metabolic mechanisms, and gene expression control (Chen et al., 2010, Beaulieu et al., 2009). Several studies on animal models (Alzheimer’s disease (AD), Huntington’s disease (HD), and Parkinson’s disease (PD) with spinal cord injury evident that lithium administration can facilitate protection against neurodegeneration. This stimulatory effect is known to endorsed via cellular and metabolic pathways that explicit effective transformation of neurotransmitters in the synaptic regions (Bosetti et al., 2002, Weerasinghe et al., 2004, Yin et al., 2006, Chepenik et al., 2010, Pavuluri et al., 2010, Narita et al., 2011). Several studies have demonstrated that lithium decreases the levels of SNAP-25 (synaptosomal associated protein-25) and MAP2 in normal rats' prefrontal cortex cells (PCC) (Lakshmanan et al., 2012). This is consistent with the documents rise in SNAP-25 in the dorsolateral prefrontal cortex (DLPFC) of individuals having bipolar I illness (Scarr et al., 2006). Existing reports suggest that the formation of numerous synaptic vesicles (SV) may elicit the root cause to the development of BD. As the synthesis of SV soly depend on the interaction between syanpsin and cytoskeletal components, determining the integrity of neural cytoskeleton underlying PCC remains an interesting area of analysis. In this context it has been established that, the cytoskeletal protein MAP2 can be a signature choice to investigate the therapautic efficacy of lithium in BD rat model system.

## 2 Methodology of literature search

An extensive review was performed to the major publications relevant to the clinical aspects of lithium dependent BD treatment effects, its mechanistic role of neurons via neuroimaging studies linked to BD patients during progressive treatment. A comprehensive literature search was conducted via Scopus, Web of Science, PubMed, and Science Direct using the keywords such as “lithium” and “bipolar disorder” and “therapeutic mechanism of action” and “clinical use” and “microtubular” and “brain imaging” and “brain structure” and “cytoskeleton” and “mood episodes” and “neuropsychiatric diseases”. Essential findings were synthesized from the literature that are futher evaluated to acquire additional publications. In order to achieve technological advancements in BD treatment, this study was passed from any timescale plans. The selection of language was restricted to English. The search obtained 485 articles, of which 330 were review publications. A manual search was conducted to all essential publications including bibliographies in order to identify any omitted articles. Articles irrelevant to the aim of the current study, language other than English, duplicate files, short-communications, magazine papers, letters, were excluded. Publications met within the inclusion criteria were screened for further analysis.

### 2.1 Cell stress in neurons, atropy and neuronal loss

Changes associated with the loss of brain structure and volume is considered to be the most devasting charasteristics features of BD pathogenesis. Several atopsy reports and brain imaging studies obtained via magnetic resonance spectroscopy [MRS] of BD subjects claimed decreased brain volume directly to the site of mood regulation. Consistent evidences suggest that this reduction in the brain density is due to neuronal/glial stress, atrophy, and death associated with neurons (Manji & Duman, 2001). A study led by Ongur and team identified substantial reduction of glial cell density (41.2 %) in the prefrontal cortex of BD subjects with a family history of mood disorders. Similar results were consisently discovered in other studies with decreased grey matter in distinct areas of the brain where emotional and cognitive processess are controlled (Brambilla et al., 2005). Such abnormalities were generally seen in the subgenual prefrontal and anterior cingulated cortex region, hippocampus, and basal ganglia of the CNS (central nervous system). Deep scanning (MRS) images revealed abnormal synthesis of N-acetyl-aspartate (NAA), myoinisotol, and choline in BD specimens. Methodologically, these are potential markers that act via neurotrophic pathways and respond during lithium administration in treatment regulations. Unlike other neurodegenerative diseases, BD is considered as a non-classic form of brain disease due to the absence of gliosis, a specific marker found in neurodegenration. However in BD, patches of possible glial loss anticipated with NAA elevation and choline reduction were common (Yildiz-Yesiloglu & Ankerst, 2006). NAA synthesis in mitochondria is associated with altered cell’s energy regulation and has been considered as a crucial parameter in neuronal stress, apoptosis, and altered gene expression that ultimately result in neurotrophic effects. Neuronal survival is facilitated by several neurotrophins including brain derived neurotrophic factor (BDNF), neurotrophin-3 (NT-3), NT-4, NT-65, NT-6, and nerve growth factor (NGF). In BD, continuous diminution of these factors strongly affect neuronal viability. Declined levels of BDNF were reported in depressive and manic episodes of human subjects that strides BDNF as a potential biomarker to identify neuronal vulnerability. Both, in vivo and in vitro studies explicit equal contribution of CREB & BDNF in mediating the therapeutic and neurotrophic efficacy of lithium. However, dysregulation of any targets of BDNF-CREB could eventually leads to atrophy to any selective subpopulation of the neurons. Therefore, deep research is needed to elucidate the kinetics behind neurotrophins mediated cell fate network during BD progression.

### 2.2 Neurotrophic effect of lithium in BD

Lithium typically stabilizes mood swings in BD with a cyclic process of administration followed by different episodes of depression. However, its effect may no longer exhibit when applicable in the reverse manner (Puglisi-Allegra et al., 2021). In a contray, lithium can less react in conditions like depressive susceptibility, drug addiction, psychotic syndromes, and fast cycling. Due to this, lithium has been advocated to treat major depressive disorder (MDD) since the late 20th century as an alternate pharmacotherapy for antidepressant-resistant individuals (Abou-Saleh et al., 2017, Undurraga et al., 2019). Currently, there is no conclusive data comparing the effectiveness of lithium monotherapy to other depressive problems such as short-term depressive episodes (Sato & Hattori, 2018). Nonetheless, numerous studies have demonstrated the beneficial usage of lithium after it has been administrated as an additional agent against depression therapy. Therefore lithium was proposed as an alternative agent to treat MDD patients who failed to respond for any antidepressants (Iwahashi et al., 2021, Sanchez et al., 2000). This effort has been proposed to constantly reduce suicidal risks in both unipolar and bipolar victims (Malhi et al., 2013) possibly acting on GABA (Malhi et al., 2013) by regulating autophagy through modulation of aggression and impulsivity (Beurel & Jope, 2014). In prophylaxis cases of manic and bipolar depression, lithium shows promising effects. This indeed pose majority of the treatment guidelines recommended lithium as the first-line therapy (Geddes et al., 2010).

On the other hand, some randomized studies based on controlled trials (RCT) to patients diagnosed with bipolar disorder I (BD-I) with depressive, manic symptoms and/or a mixture of both were reported to have significant increase in the recurrence rate when compared to treatments involved with placebo (Weisler et al., 2011). Conversely, open labelled randomized trial studies to BD-1 patients with no longer chronic episodes underwent long-term treatment to prove high relief on lithium-monotherapy when compared to that of valproate monotherapy (Geddes et al., 2010). These observations indicate the prophylactic efficacy of lithium to treat manic conditions than that of depressive episodes (Curran & Ravindran, 2014). Several studies have suggested that lithium can control bipolar psychosis via BD which is often associated with manic episodes and psychotic features (Fornaro et al., 2016). However, limited studies were conducted for systematic assessments on lithium to treat both mania with psychosis. Comparative studies of various neutoleptics against lithium based treatment to I and II-level episodes of mania and psychosis revealed chlorpromazine as an effective medication to treat psychotic features (Prien et al., 1972). Similarly, the drug sripiprazole was also promising than lithium and placebo in treating psychotic features (Keck et al., 2009), where lithium was found effective as that of quetiapine and superior than placebo in treating psychosis with manic episodes (Bowden et al., 2005).

Lithium monotherapy is considered to have high significance than placebo to treat manis in psychotic subtype (Swann et al., 2002). It also produce early improvements in psychotic symptoms, and exhibit similar efficacy to both psychosis and non-psychosis mania (De Sousa et al., 2012). It has been long recognized that lithium can modulate immune responses and explicit unique features to empasis affective disorders by altering proinflammatory cytokine synthesis and through GSK-3β inhibition (Rybakowski et al., 2018). In addition to acute clinical effect of lithium, it is assumed that long term treatments could enhance clinical effects such as inflammatory suppression, neuroprotection of brain cells, and prevention of depressive episodes. Studies using structural neuroimaging and MRI analysis revealed close association between lithium treatment that had increased grey matter volume of the affected victims than untreated patients (Phillips et al., 2008). In a similar study, examinations among 17 lithium treated and 12 untreated BD patients showed significant increase in the quantity of white and grey matter (Bearden et al., 2008). Other clinical reported have showed enhanced grey matter density in the cortical regions of the brain that had a greater volume of hippocampal in lithium-treated patients. Specified evaluations on potential targets such as NAA and myoinositol followed by four weeks of lithium administration resulted increased NAA levels in distinct regions of the brain and reduced levels of choline and myoinositol (Silverstone et al., 2003). This suggest that lithium has a strong neurotrophic effect on BD patients. One of the most replicating findings identified significant reduction of anterior cingulate volumes of the brain of BD patients compared to their healthy volunteers (Sassi et al., 2004).

### 2.3 Lithium toxicity and adherence

Despite the global importance of lithium as an effective therapeutic substance, its direct usage is restricted due to the growing margin of toxicity (Gitlin, 2016). Self-administration of lithium often results increased dose index that emphasize nearly 20–27 % hospitilzed poisoning and stimulated death rates of 10–25 % (Camus et al., 2003). Dose dependence of lithium may therefore vary among individuals and can be determined by indexing the pharmacokinetics and risk of intoxication. Therefore the recommended usage of lithium is calculated based on the difference between its excretion rate in individuals and the percentage of serum concentration levels. Usually, 10–80 mmol with a plasma concentration of 0.4–1.2 mmol/L is recommended for effective treatment practices (Couffignal et al., 2017). Increase in the serum concentration (<1.2 mmol) is considered potentially lethal and life threatning. Care should be taken during lithium administration and periodic monitoring of blood serum levels will ensure safety from adverse effects. Another persistent problem associated with lithium administration is non-adherence of patients to the prescription. It is expected that discontinuation of lithium intake leads to adverse effects (Table 1). Henceforth, new strategies must be developed to improve regular followups to ensure unnecessary termination of lithium treatment.

**Table 1:** Chronic effects of lithium (Drug doses).

**S.No**	**Chronic effects common** **(< 10 %)**	**Lesser common chronic effect (>10 %)**	**Reference**
	Weight gain Polyuria Thirst Diarrhea Tremor	Reduced emotional and /or perceptual intensity^a^ Impairment of Cognitive^a^ Acne Nausea^a^ Psoriasis Hypothyroidism^a^ Kidney impairment^b^ Cardiac arrythmia Hyperparathyroidism	(Mendiola et al., 2021)

a Dose related

b Total lithium represents the availability of lithium over time and its serum-lithium concentration to the drug-dose level

### 2.4 Molecular mechanism of lithium

**Fig. 1:**
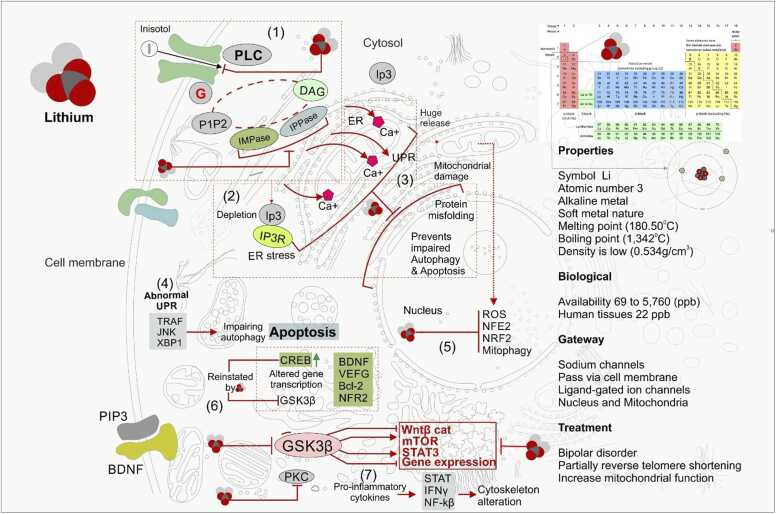
Summarize the molecular events that takes place by lithium in specific cell target.

Lithium is known to modulate multiple cellular pathways but the exact mechanism underlying has not been fully elucidated. Several studies have suggested that lithium can specifically target neurological events to enrich neuroprotection and modulate mood stabilizing activities (Malhi et al., 2013). However, various hypotheses explicit that lithium action can determine cell-fate and drive glycogen metabolism to confer BD treatment (Price and Heninger, 1994, Goodwin and Jamison, 1990, Berridge et al., 1989, Avissar et al., 1988). In this, the inositol depletion hypotheses model was highly accepted among all mechanisms where lithium inhibits inositol uptake and prevents IMPase (inotitol monophosphatase) and IPPase (Inositol polyphosphate-1-phosphatase) resulting in IP3 depletion of cells that acts as an endogenous source of inositol (Berridge et al., 1989, Avissar et al., 1988, Maeda, 1970). Cells that are incapable to receive external signals fail to produce InsP3 (inositol 1,4,5 triphosphate) and therefore block the InsP3-dependent response (Fig. 1). This stimulates ER stress to release huge quantity of Ca^2+^. Lithium inhibits apoptosis and autophagy linked to ER stress, deposit dysfunctional mitochondria and prevent proteins from misfolding. This results accumulation of ROS leaked from mitochondria that downregulate the nuclear factor erythroid 2 (NFE2) favoring mitophagy and mitochondriogenesis. Abnormal unfolded protein response (UPR) upregulates TRAF/JNK, and XBP1favoring apoptosis during autophagy process, whilst, lithium triggers downstream of these pathways by inhibiting PKC and GSK3β via series of pathways. Lithium facilitate GSK3β inhibition and impair neurotrophic transcription, neuroprotective, and inhibit the expression of antioxidant genes (NRF2, Bcl-2 [B-cell lymphoma 2], VEFG, and BDNF), to produce proinflammatory cytokines via STAT, INFℽ, and NF-kβ activation to reconstruct the altered cytoskeleton.

In the other way, the GSK-3 mechanism that regulates synaptic plasticity and cell-apoptotic events favor the progression of cell-death. Therefore, the attenuated characteristics of this pathway might regulate cellular resilencing and neuroplasticity (Carter, 2007). Any alteration to the GSK-3 mechanism might affect the antimanic and antidepressant activity via gene regulation (Martinowich et al., 2009, Serretti et al., 2008, Lee and Kim, 2011). Hence, lithium stimulation can influence various neuroprotective pathways by increasing the GSK3β phosphorylation and migating the effects of excitotoxicology (Table 2) (De Sousa et al. 2015). Studies have identified that lithium can respond positively via GSK3β gene expression pathway and phosphorylation to control the BD symptoms. In addition, lithium can directly inhibit GSK3 activity and indirectly affect Akt activation pathway (Iwahashi et al., 2014). This action will suppress the behavioural effect of DA transmission via β-arr2-mediated Akt/GSK-3 signaling mechanism. These evidences suggest that the therapeutic effect of lithium anticipate sensitization to BD subtype in the similar way to its phenotype (De Sousa, Streck, et al., 2015). Changes occur in the intracellular calcium levels might play significant role in modulating neurotransmitter transmission in the synaptic region. This is associated with mitochondrial function to explicit neuroplasticity, calcium balance, and apoptosis activity (Neher & Sakaba, 2008). However, a dysregulated oxidative metabolism could rely on mitochondrial damage and favor destruction of genetic material that eventually leads to neuronal apoptosis (Gigante et al., 2011, Konradi et al., 2004). Similarly, the dysfunctional mitochondrial samples of BD patients show expression of hippocampal genes associated with destructive mitochondrial proteins (Konradi et al., 2004). In addition, enhanced telomere shortening occurring in BD patients have great influence due to stress-related oxidative damage (Simon et al., 2006). In fact, several cellular mechanisms associated with lithium favor antioxidant expression (Macedo et al., 2013), are known to suppress the assembly of stress proteins (Nciri et al., 2013), delay in the synthesis of proinflammatory substances (Wang et al., 2013, Watanabe et al., 2014, Myint and Kim, 2014, Kim et al., 2007), and affect gene expression associated with oxidative cytoprotection (Rizak et al., 2014). Several observations declare that administration of lithium in BD patients could results reduced lipid peroxidation levels (De Sousa et al., 2014), enhanced mitochondrial function and reverse the stimulation of oxidative stress pathways (De Sousa, Streck, et al., 2015). It is expected that lithium can also associate with secondary messenger factors to enhance its therapeutic potential via pathways including protein kinases C, phosphoinositide cycle, and intracellular calcium production (Malhi & Outhred, 2016). Lithium ameliorate multiple fuctions such as ameliorating mitochondrial dysregulation via inositol depletion and inhibiting IMPA1 (Sarkar et al., 2005), and provoke neuronal plasticity by blocking protein kinase ‘C’ (Tsui et al., 2012). Lithium can downregulate TRPC3 (transient receptor potential channel 3) to maintain Ca^2+^ homeostasis (Zaeri et al., 2015) and stimulate c-AMP pathway (Heinrich et al., 2013). These observations infer lithium as an effective therapeutic agent to act upon several cellular pathways to stabilize neuroprotection in BD patients. (Table 3, Table 4)

**Table 2:** Shows increased risk of lithium intoxication and its associated signs and symptoms.

**Signs and Symptoms**	**Risk Situation**	**References**
**Light intoxication**: Nausea, tremor dysartria, lethargia, cognitive impairment	Increased lithium concentration elevate water and salt loss (e.g. increased sweating) and infect gastrointestinal system. Changes in the levels of water and salt	(Gitlin, 2016)
**Average intoxication**: Vomiting, muscle twitching, impaired walking pattern, and disorientation.	intake indicate disease condition. Elevated lithium elimination via kidneys indicates old age, and/or kidney dysfunction.	(Gitlin, 2016)
**Chronic intoxication**: Convulsions, ataxia, delirium, kidney dysfunction	Nonsteroidal anti-inflammatory drugs and/or concurrent treatment with thiazide	(Haussmann et al., 2015)

^a^ Serum lithium level appear with above 1.2mEq/L. Since CNS secreates less lithium concentration compared to that of serum levels, lower range of lithium exhibit incompatible to intoxication.

**Table 3:** Differential protein expression in rat models after lithium administration.

**Spot No.**	**Protein name**	**Accession No. (NCBI)**	**Gene**	**MW (Da) (pI)**	**MASCOT**	**Fold change**	***p*****-value**	**References**
***Proteins with increased levels***								
10	Hexokinase Type I, Chain B, rat brain	GI5542104	*Hk1*	102,455.2 (6.29)	222	1.31	0.00023	(Plenge, 1982)
22	α Synuclein	GI9507125	*Snca*	14,506.2 (4.74)	280	1.34	0.006	(Perez et al., 2002)
11	TNF receptor-associated protein 1	GI55741837	*Trap1*	80,410.8 (6.56)	175	1.26	0.025	(Liu et al. 2010)
24	Endosulfine α	GI9624979	*Ensa*	13,326.7 (6.62)	128	1.26	0.016	(Plenge, 1982)
21	Proteasome (prosome, macropain) subunit, β type 2	GI34849630	*Psmb2*	22,897.7 (6.96)	100	1.27	0.014	(Sasaki et al., 2010)
***Proteins with decreased levels***								
25	EGF-containing fibulin- like extracellular matrix protein 2	GI53733803	*Efemp2*	44,820.3 (4.79)	34	−1.78	0.046	(Huang et al., 2020)
19	Synaptosomal-associated 25kD protein	GI57114057	*Snap25*	23,300.2 (4.66)	212	−1.70	0.034	(Etain et al., 2008)
1	2'−5′ oligoadenylate synthetase 1 H	GI57222310	*Oas1h*	42,800.7 (5.38)	40	−1.49	0.024	(Soveg et al., 2021)
12	Calreticulin	GI11693172	*Calr*	47,965.8 (4.33)	344	−1.47	0.017	(Iwahashi et al., 2021)
_2_*	Microtubule-associated protein 2	GI547890	*Map2*	202,287.6 (4.77)	102	−1.45	0.007	(Sanchez, Diaz-Nido, et al., 2000)
_3_*	Microtubule-associated protein 2	GI56625	*Map2*	198,445.6 (4.76)	225	−1.38	0.036	(Sanchez, Diaz-Nido, et al., 2000)
23	Similar to diphosphoinositol polyphosphate phosphohydrolase	GI27704734	*Nudt3*	19,083.6 (6.00)	35	−1.38	0.0011	(Shears, 1998)
15	Laminin receptor 1/ Ribosomal protein SA	GI8393693	*Rpsa*	32,803.4 (4.8)	203	−1.36	0.04	(Lithgow et al., 2020)
17	Clathrin, light chain B	GI16758690	*Cltb*	25,102.1 (4.56)	268	−1.35	0.015	(Brodsky, 2012)
13	Growth associated protein 43	GI8393415	*Gap43*	23,589.3 (4.61)	39	−1.35	0.035	(Laux et al., 2000)
9	Brain abundant, membrane attached signal protein 1	GI11560135	*Basp1*	21,777.4 (4.5)	69	−1.32	0.0097	(Korshunova et al., 2008)
7	Valine t-RNA synthetase	GI484949	*Vars*	66,744 (6.19)	41	−1.32	0.024	(Friedman et al., 2019)
16	2',3′-cyclic nucleotide 3′-phosphodiesterase	GI294527	*Cnp*	47,238.6 (9.03)	47	−1.29	0.012	(Sprinkle, 1989)
18	Glyceraldehyde- 3-phosphate dehydrogenase	*GI8393418*	*Gapdh*	35,805.2 (8.14)	151	0.0038	−1.27	(Dar et al., 2021)

**Note:** NCBI (National Center for Biotechnology Information); MW (Da) Molecular Weight, Dalton; MASCOT (Name of a software mainly used for protein identification)

**Table 4:** Proteins classification and it functions that are differentially expressed in chronic lithium treatment.

**Spot**	**Proteins**	**Location of the cell**	**Biological function**	**References**
**Snaptic**	**Snca**	Cell membrane, cytoplasm, nucleus	Regulates insulin secretion; Synthesis of neurotransmitters and uptake; Facilitate synaptic to vesicle docking during exocytosis; Synaptic transmission cascade;	(Jewell et al., 2010)
	**Cltb**	Cell membrane	Facilitate transportation of intracellular proteins and vesicles.	(Royle, 2006)
	**Snap25**	Cell junction synaptosome, Plasma membrane, cytoplasm	Response to ℽ-interferon; presynaptic signals coupled with membrane traffic; dopamain synthesis and regulation; downregulation of mono oxygenase.	(Giovanni et al., 2012)
**Signaling**	**Nudt3**	Cytolsol	Cell-cell signaling; turnover of inositol pyrophosphates. Diphosphoinositol-polyphosphatediphosphatase activity;	(Gu et al., 2017)
	Gap43*	Synaptosomes	Nervous system development; growth cone guidance; Actin dynamics. Calmodulin binding; Glial cell differentiation; Axon choice point recognition; Activation of G-protein coupled reaction and Protein kinase-C signaling pathway.	(Denny & John, 2006)
	**Oas1**		Antiviral property of interferon.	(Kristiansen et al., 2010)
	**RPSA**	nucleoli ribosome, cytosol, cellsurface	Ribosome binding; translation and elongation; cell-matrix attachment; activation of receptor; ribosome homology analysis.	(Nilsson et al., 2004)
	**Calr**	Cytosol	Ca^2+^ binding; Transcriptinal regulation and apoptosis.	(Michalak et al., 1999)
**Cytoskeleton**	Map2**	Cytoplasm, cytoskeleton	Calmodulin binding; structural molecule; negative regulation of microtubule depolymerization;	(Johnson & Jope, 1992)
	**Basp1**	Plasma membrane	Neuron-specific regulates neurite outgrowth; Calmodulin binding; component of brain lipid raft;	(Biskup et al., 2016)
	**Efemp2**	Extracellular space, Basement membrane	Calcium ion binding; Protein binding and membrane activity; Structural aspects of extracellular components.	(Halabi et al., 2017)
	**Cnp**	Cytosol	RNA metabolism; Organization of cytoskeleton; Synaptic transmission; Phosphodiesterase action.	(Biskup et al., 2016)
**Metabolic**	**Psmb2**	Cytosol and nucleus	Ubiquitin mediated protein digestion	(Halabi et al., 2017)
	**Trap1**	Cytosol, mitochondria.	Protein folding; ATP binding; chaperone activity.	(Raasakka & Kursula, 2014)
	**Hk1**	Cytosol, Mitochondria.	Glycolysis; Anti-apoptosis.	(Hochstrasser, 1996)
	**Gapdh**	Perinuclear region, cell membrane, cytosol	Glycolysis; energy metabolism. Glucose metabolism; oxidation reduction; apoptosis;	(Masuda et al., 2004)
	**Vars**	Cell nucleus	Valyl-tRNA biosynthesis	(Liu, Hu, Agorreta, Cesariom, et al., 2010)
**Transport**	**Ensa**	Cytoplasm	Ion channel inhibitor activity; transport; response tonutrient; receptor binding	(Kubota et al., 2020)

**Note:** *Spot 2 represents a phosphorylated form of Map2.

### 2.5 Lithium mediate direct-targets of neuroprotection

#### 2.5.1 Glycogen synthase kinase 3 (GSK-3)

GSK-3 is a serine kinase that remains one of the most important targets of BD pathophysiology and therapeutics due to its enormous involvement in distinct cellular pathways such as apoptosis, synaptic plasticity, gene transcription, glycogen synthesis, resilence, and regulating circadian cycle (Jope, 2003). When activated, GSK-3β functionally triggers cyclic AMP response element binding protein (CREB), βcatenin, and other transcriptional factors. Signals that arise from a number of other pathways such as the Wnt pathway, protein kinase A (PKA), PI-3kinase, and protein kinase C (PKC) can directly regulate GSK3 activation. Although GSK3 can be activated via several pathways, some isoforms of PKCs critically inactivate GSK-3β. However, its functional activation or inhibition can be determined by changing targets of various signaling pathways in the neuronal system. Notably, when Wnt pathway activates β-catenin (transcriptional factor to regulate memory consolidation) the expression of GSK3 hinders. Other targets of GSK3 includes MAP, cell-cycle mediators, and metabolic regulators. GSK3 can directly control various neurotransmittor systems such as dopaminergic, serotonergic, and glutamatergic (Beaulieu et al., 2009). It is identified that various neurotrophic effects are closely associated with GSK3 inhibition. Its inactivation could directly influence gene transcription, block apoptosis and maintain structural stability of cells (Chin et al., 2005). Lithium downregulate GSK3 to safeguard neuronal cells against injuries caused by cellular responses. Specifically, the inhibition constant (K_i_) at which lithium inhibit GSK3 is 1–2 mM (serum therapeutic scale, 0.6 – 1.2 mM) (Stambolic et al., 1996). Rats treated with lithium significantly enhances cytosolic β-catenin and in mouse models it increases brain β-catenin dependent transcription and reduced the synthesis of amuloid-β peptide (O’Brien et al., 2004). Demonstration of knockout mice models resulted consitent hypothesis that lithium mediate direct inhibition of GSK3 via enhancing the levels of β-catenin (Gould et al., 2004). New approaches towards the identification of effective GSK3 inhibitors are currently in different stages of development.

### 2.6 Action of lithium on cytoskeletal proteins

In a general perspective, many drugs used today to treat neurodegenerative diseases target cytoskeletal proteins to modulate NDs. However, some data suggest that these neural proteins are highly associated with neuronal degeneration and cellular dysfunction (Beaulieu et al., 2015). It is expected that the regulation of Ca^2+^ misguide neuronal cytoskeleton that results cell injury and cause damage to various regions of the CNS. These regulations disrupt cell signaling and dissociate cytoskeleton organisation to form morphological changes in the primary culture plates (Pierozan and Pessoa-Pureur, 2017). Therefore the cytoskeletal polymers take part pleiotropic effect to neuronal morphogenesis and affect intracellular mobility that further influence pre and post-synaptic effects. This eventually results microfilament abnormalities and form neuronal dysfunction (Kinoshita et al., 2012). Proteins that belongs to the synaptic family tend to attach with the cytoskeleton and synaptic vesicles to inhibit subsequent movement of it to the pre-synaptic region and blocks the synthesis of neurotransmittors (Hilfiker et al., 1999). The usage of cytoskeleton framework to neuronal cells play structural dynamics of long axons and dendritics. Their rigidity confers strong electromagnetic input for long distance travel and established organizational necessity for synaptic transmission (Tischfield & Engle, 2010). Disintegration of microfilaments has been inferred in multiple NDs such as the BD. Microtubules are cytoskeletal structures made from globular proteins that assist cellular locomotion and cell shape determination. It consists of hollow fibers composing heterodimeric forms (25 nm in diameter) of α and β tubulin proteins lined up to form cylindrical lattice (Conde & Caceres, 2009). After synthesis, post-translational modifications determine the stability and shape of the microtubule (Ikegami & Setou, 2010). Notably, some microfibers undergo additional stability via reducing depolymerases activity before assembled to form 3D structures (Peris et al., 2009). Acetylation and detyrosination are two such modifications enrolled by microtubules of the neurons (Gache et al., 2010). Any alteration in the interaction between synapsin and cytoskeletal components can interfere with synaptic vesicle formation, MAP2 and thus leads to BD. In general, two types of MAPs exist: structural and end binding +TIP proteins. Of this, structural MAPs support microtubules for assembly and +TIP proteins stimulates microtubular growing edges to form linkages with other protein complex (Mohan & John, 2015 of 21). Persistant inflammation to the brain cells results synaptic pruning and bring structural changes that results loss of synaptic density and affect cognitive functions (Rosenblat et al., 2015). MAPs are group of proteins that are associated with microtubules of neurons to promote neuronal stability. MAP2 is highly present in the brain and its functional role in the regulation of neuronal plasticity has been long-studied. Since MAP2 act as core regulators along with the microtubular proteins, distinct neuropsychiatric disorders are associated with their dysregulation. Different isoforms of spilced MAP2 evolved from multiple exons were identified that can be further divided into high and low molecular weight MAP2s that are expressed in the neuronal cells and/or glial cells. Usually MAP2 bound to the surface rim of the microfilament facilitate attachment to its neighbouring microtubule to form stable bundles. This characteristic role of MAP2 is to form nucleation factor for tubulin polymerization. Studies using mice models revealed elevated brain development and affected dendritics elongation in the absence of MAP2. Therefore reduced dendritic spine density and arborisation can highly contribute to various neuropsychiatric disabilities.

The concept that dysregulated cytoskeleton anomalies in neuronal architecture in BD subjects are further evident via animal models and clinical studies. Prominent changes associated with tubular dysfunction were often found in regions of prefrontal cortex of the affected individuals resulting atrophy in chronic condition. Notably, loss of synapses dysregulates mood homeostasis that leads to distorted feedback loops suggesting a loss in MAP2 maintaining spine stability. As MAPs serve as a biomarker in determining neural stability their influence can be measured via blood and CNS fluids (Maccioni & Cambiazo, 1995). During synaptic dysfunction, MAP2 influence CREB that hinder PKA anchoring to dendrities (Harada et al., 2002). However, CREB expression regulates BDNF that play central role in maintaining synapse health. Immunohistochemical analysis revealed substantial reduction of hippocampus and prefrontal cortex area in several post-mortem specimens in patients with NDs which is accompinied by loss of primary and secondary basal dendrities; whilst, a 28 % loss was reported in BD patients (Bouras et al., 2001 of 20). Considering these findings it is evident that the mRNA of MAP2 was intact in the hippocampus region and the reduction in the immunoreactivity is due to the phosphorylation of MAP2 that hinders the epitope region of the microtubule. This alteration eventually contribute to dendrite loss and results reduced synaptic area to further affect information processing in most psychiatric conditions. Given that MAP2 is a highly available brain protein, aberrant phosphorylation by kinases can contribute to various forms of psychiatric illness. Importantly two MAP2 kinases ‘calcium calmodulin kinase II (CAMKII), and glycogen synthase kinase-3 (GSK-3) can phosphorylate the tubulin binding domain of microtubules forming decreased binding ability that alternatively reduces the microtubular stability (O’Leary and Nolan, 2015 of 20). Similar to MAP2 is MAP6, a STOP (stable tubule only polypeptide) protein highly found in the neuronal cells of the brain mostly in its spliced form. Both MAP2 & MAP6 stabilizes binding capability of adjacent microtubules via calmodulin regulation (Lefe`vre et al., 2013). Mice model studies of MAP6 gene deletion exposed wide range of phenotypic features that includes impaired cognitive function, hyperactivity and dysregulated serotonergic function related to synaptic connectivity.

Synaotosomal-associated protein (SNAP-25) is a 25 kDa component of the SNARE protein complex which has central role in synaptic exocytosis and can directly interfere with different calcium channel regulation when adhered with syntaxin-1 and synaptobrevin. Inhibition of SNAP-25 strongly affect synaptic transmission as it involves in synaptic vesicle trafficking. SNAP-25 is also present in the growth cones of neurites extension during the formation of synaptic connections (Jacobsson et al., 1996). Analysis of various brain samples of humans and animals after a trauma revealed elevated SNAP-25 levels. Both preclinical and clinical observations led an idea that SNAP-25 could be considered as an effective biomarker to determine neurodegeneration in adolescence as the levels may alter due to hormonal changes (Kendler et al., 1999 of 24). The expression of SNAP-25 can be affected by utero virus attack and their changing levels may serve ideas of viral etiology to schizophrenia and bipolar disorder (Wright et al., 1995). Several studies have reported that the occurance of polymorphism in the *Snap-25* gene can be associated with the early onset of BD (Etain et al. 2010). It is identified that some polymorphisms of *Snap-25* gene can alter specific traits of the neurodegenerative diseases and change the behavioural aspects of healthy individuals. Moreover, several single nuecliotide polymorphisms of this gene (*i.e.* rs363043, rs353016, rs363039, & rs363050) were identified to associate with Intelligence Quotient (IQ) phenotype in healthy individuals. SNAP-25, syntaxin, synapto brevin and synaptophysin were identified to help the docking capability of synaptic vesicles with neurotransmittors; whereas, neuronal cytoskeleton plays crucial role in maintaining axon elongation and dendrites differentiation. Neuronal polarity as determined by various molecular events of axon and dendrites differentiation takes place in normal condition where potential expression of SNAP25 occurs (Pierozan, 2017). Some studies of autopsy CNS samples of dorsolateral PFC of bipolar disorder type I patients revealed the presence of increased concentration of SNAP25 (Kinoshita et al., 2012). However, in lithium treated samples the concentration of SNAP25 was decreased to 1.7 fold suggested that the potential action of lithium towards glutamate-induced excitoxicity in BD victims. On the other hand, Cltb (integral component of clathrin vesicles) levels declined to 1.35 fold after lithium treatment which reflect to a reduced neurotransmittor receptor levels and synaptic vesicle exocytosis. Efforts from Genome wide association studies (GWAS) failed to identify SNAP-25 gene to positively correlate with BD development; whilst, some case controlled studies towards the autopsy tissues led varience of SNAP-25 (*i.e.* SNAP-25b) associated with BD (Etain et al., 2010). Increasing analysis by several study groups further reported decreased quantities of SNAP-25 in the stratum oriens and high levels in the subiculum of the hippocampus (Fatemi et al., 2001).

Protein kinases and phosphatases are well-known neuronal mechanisms that undergo modifications to facilitate effective control to neuronal cytoskeleton-network (Qvit et al., 2016). This mechanism can associate with microtubular proteins that influence the plasticity of dendrites and axons via response from extracellular signals (Guo et al., 2010). MAP2 which is a high molecular weight compound that undergo phosphorylation and dephosphorylation to take part in dendrites development and synaptic plasticity. Some findings claimed that the microtubule associated aminoacid ‘Threonine’ (1620/1623) undergoes phosphorylation to become ‘Proline’ and hence become targets for protein kinases (Guo et al., 2010). Specific antibody studies using ‘Ab-305’ observed reduced phosphorylation to MAP2 in cultured cerebellar neurons. However, the effect of phosphorylation of MAP2 could be blocked by lithium in limited-time cultured neurons suggesting the influence of lithium to glycogen synthase kinase-3 (Guo et al., 2010). Moreover, it has been observed that the levels of cytoskeletal network of Schizophrenia and BD are often affected by protein damage. Determining the levels of cytoskeleton alteration coupled with neuronal functioning may help to identify their mechanistic role in BD progression (Qvit et al., 2016). A significant reduction of ndufv2 (NADH dehydrogenase [ubiquinone] flavoprotein-2) stimulate both polar and bi-polar transformation during *in-vivo* and *in-vitro* studies. Besides this, ndufv2 affect tubulin stability and promote breakdown of actin fibers in cortical neurons (Von Stetina et al., 2008). This failure affect the intracellular segments of neurons to alter neuronal factors, and axonal retraction via initiating secondary level damage to brain tissues causing serious inflammation (Chen et al., 2015). It is observed that Cdk5 and/or GSK3β play crucial role during the restoration of neuronal microtubules; however additional studies are needed to understand the entire mechanism behind this process. It is postulated that phosphorylation of collapsin response mediator protein 2 (CRMP2) via Cdk5/ GSK3β exhibit relapsed neuronal cytoskeleton resulting stunded axonal growth (Guo et al., 2010); whereas, CRMP2 without polymerization could stabilize microtubule proteins within the neuronal cells of the BD patients (Chen et al., 2015). Site-specific modulation of MAP-2 protein in rat brain culture models influenced the proline rich domain of the neuronal microtubular protein (Sanchez et al. 2000), initigating that maintaining the stability of neuronal cytoskeleton can potentially restore neurodegenerative problems (Nagai et al., 2016). Lithium also shows positive effect via PKC activation and G-protein coupled reaction in BD patients (Tobe et al., 2017). Thus lithium is known to modulate gene control in BD patients (Benitez-King et al., 2004) by focusing on rapid developments in GSK-3β, β-cathening, and neurotrophin cascade based controls (Hahn et al., 2005). Some condition of acute BD treatment involves combined medication of lithium and antipsychotics where lithium alone can be ineffective with anticonvulsants. However, the administration of adjunctive antidepressants exhibit limited usage during increased depressive condition (Fatemi et al., 2009). Microarray gene expression profiling of mRNA in mouse models of brain examination treated with lithium carbonate enrich visibility to assess mood stability disorders (Chetcuti et al., 2008, Sani et al., 2017, Wada, 2009). Furthermore, the relationship of AKT-1 gene variants in NDs would bring translational research outcomes to safeguard neuroprotection (McQuillin et al., 2007).

### 2.7 Mitochrondrial stress regulations of lithium

The pathophysiology of BD comprises loss of neuronal cells due to apoptosis driven by diverse singaling pathways. Increased oxidative stress and apoptosis are two known parameters of BD regulated by mitochondrial NAA and Bcl-2 mediated by intracellular calcium levels via ER receptors like IP_3_, and Bcl-2. The release of calcium is a general phenomenon by cells and any alteration to this can cause serious damage to the cellular mechanary. In the neural cells of BD patients calcium continuous to flow towards the mitochondrial cavity causing dysregulated adenosine triphosphate (ATP) synthesis that results the release of cytochrome ‘c’ factor to provoke apoptosis. This indeed triggers mitochondria to synthesis free radicals (ROS) causing oxidative stress and affect cellular antioxidant glutathione to be reduced in BD subjects. Several research outcomes elucidated that the end product of thiobarbituric acid reactive substances (TBARS), lipid peroxidation, antioxidant enzyme activity (superoxide dismutase [SOD] and catalase [CAT] were increased in BD patients as a result of increased oxidative stress in the mitochondria (Machado-Vieira et al., 2007). Lithium incorporation can specifically target ER and mitochondrial proteins result in the elevated levels of Bcl-2. It also induce ER chaperones proteins calreticulin, GRP94, and GRP78 to safeguard misfolded proteins against deleterious effects. Importantly, lithium blocks the synthesis of IP_3_ to import neuronal growth-cones. High concentrations of lithium can directly change oxidative damage to lipids and proteins and increase the levels of glutathione s-transferase (GST) and induce DNA fragmentation (Shao et al., 2008). However in BD patients, optimized levels of lithium usage can gradually enhance CAT and TBARS to exhibit antioxidant ability.

### 2.8 Regulation of protein authophagy in BD

The equlibrium of protein homeostasis is ubiquitious in the cellular system where removal of impaired protein components could be accomplished by cellular processess (Kerr et al., 2018). Lithium intrigunigly act upon dysregulated and misfolded proteins of the neural system to enrich the degradation process thus regulating proteasomal activity and autophagy. This breakdown of protein aggregates in the neural system synthesis homeostasis and modulate proper functioning of neurons. Proteins that are known to be involved in the aggregation process are α-synuclein and huntingtin (Motoi et al., 2014). Studies have claimed that lithium can induce autophagy via depletion of free inositol and reduces IP_3_ concentration by blocking IMPase and inositol transporters. This effect of lithium-dependent neuroprotection were identified via animal models characterized by misfolded proteins accumulation.

### 2.9 Challenges and myths around lithium drug

In the recent years the prescription of lithium had gradually declined internationally. A general survey conducted by the US national market during 2002 – 2003 found that lithium as the initial drug to be prescribed to 7.5 % patients with BD. However, during 1997 – 2016 the utility of lithium as a main drug has reduced to half in percentage according to the survey conduced by National Ambulatory Medical Care Surveys (Rhee et al., 2020). One among the major reasons to this downward trend is the availability of other drugs such as antipsychotics and antiepileptics (Malhi et al., 2023). In recent years several myths regarding lithium prescribtion were dispelled due to growing evidence based treatments has increased (Malhi et al., 2023). Notably in general, lithium is a drug of maintainance choice to treat BD and is argued to have acute and prophylactic effects when used. Some evidences suggests that the effects of lithium is far larger than any other drugs (Nestsiarovich et al., 2022). Randomized controlled trials of lithium with other drugs such as valproate, lamotrigine, olanzaping, and quetiapine confirmed that it could be the primary choiced drug to relapse the BD patients. Several systematic reviews claimed that lithium monotherapy had improved outcomes when compared to other mood-stabilizing monotherapy drugs that includes valproate, lamotrigine, olanzaping, and quetiapine. As for this concern, the long-time use of lithium gives renal and thyroid problems. Therefore long-time use of lithium requires careful evaluation and patients with no effect should be discontinued. Longitudional studies have reported that the possibility of chronic renal problems can be avoided via proper monitoring of serum creatinine (every 3–6 months) levels and via managing serum lithium levels to 0.6–0.8 mmol/L (Kessing et al., 2015c). Similarly, stimulation of hypothyroidism is not a part of lithium treatment rather some part of it results due to surveillance bias and frequent testing of thyroid during BD treatments (Lambert et al., 2016). Some of the recent challenges that likely hinder research on BD are (1) Changes associated in the diagnostic protocols across psychiatric centers, (2) Obtaining less clinical experience per clinician due to low BD patients, (3) Difficulty to recruit group-dependent psychoeducaton on a regular basis, and (4) Conduction of limited research.

### 2.10 Therapeutic intervention target microtubule

Oxidative stress is considered to be one of the major interventions that cause cytoskeletal damage. Accumulation of free radicals in the microtubular region destabilize the strength and result loss of polarization. This destructive signs eventually activates apoptotic signaling that leads to neuronal death. Several studies have claimed that activation of cellular antioxidants could improve neuronal function during cytoskeleton disintegration by improving dendrite stability and microtubular polymerization. However, the usage of an effective pharmacological substance against BD is challenging as sepcific research remains largely experimental. In this context, approaches towards the usage of artifically synthesized bionic microtubules are proposed to ensure restructuring defective microtubules (Woolf, 2009). Samples isolated from olfactory biopsies revealed perturbations in the microtubule organization of BD patients. Neuronal cells of BD patients exhibit characteristic changes in the cytoskeletal framework that are associated with the disorder (Solis-Chagoyan et al., 2013). Various therapeutic practices ameliorate the efficacy of neurosteriods in the management of depressive symptoms. They were initially identified as microtubular binding partners synthesized de novo from cholesterol in the nervous system that play povotal role in neuroprotection and provide long-time memory (Plassart-Schiess & Baulieu, 2001). To date, lithium is considered as an effective antidepressent drug as it directly involves in a wide range of molecular targets. The results of a gene enrichment study obtained from 7000 BD patients and their controls indicated the association of genetic disruption in microtubular pathways (Drago et al., 2016). However, lithium acting on GSK-3 inhibit tau and MAP1B phosphorlation to facilitate microtubule remodelling. This method rebuilds cytoskeletal framework and restore neural plasticity in depressive disorders. Notably, enhanced research validation is required to ascertain whether restructuring disrutped microtubules could be a therapeutic possibility of lithium in the complex CNS environment (Cole, 2013). Despite all, this study indicates the potential impact of neurodegenerative syndromes relative to mood disorder with special emphasis on bipolar disorder. The results of several clinical studies revealed genetic relationship between key regulators of cytoskeleton maintainance and diminished MAP2 that offer a hallmark to several neurological problems. Perhaps it is clear that microtubules are linked to neuronal polarization and involved in capturing synaptic connectivity.

## 3 Discussion

For decades, lithium has been used therapeutically to treat bipolar disease (BD), mostly because of its proven effectiveness in mood stabilization and lowering suicidal risk. Emphasizing its molecular and cellular actions to the dysfunctional neurons of the CNS, this review synthesise translational data supporting several neuroprotective mechanisms of lithium. Observations from key molecular studies such as proteomic, genomics, and neuropharmacological research anticipate that lithium can act to several regulatory nodes, profoundly affecting cytoskeletal integrity, synaptic plasticity, neurotransmitter release, and oxidative stress responses. The mechanism through which lithium acts is by blocking IMPase to lower inositol levels and thereby inhibit phosphoinositide signaling. Many people agree that this ancient route explains how lithium stabilizes mood. However, new studies highlight a more complicated interaction involving GSK-3β inhibition which can modulate as a critical agent to many cellular processes including inflammation, neuroplasticity, and GSK-3β death signs. Lithium enhances serine-9 phosphorylation of GSK-3β to promote neuronal survival rate and relapse synaptic resilience therefore reducing the realiability of neurodegenerative processes seen in BD. Proteomic data generated from the prefrontal cortex of lithium-treated animal models revealed significant changes associated with cytoskeletal proteins including MAP2, SNAP-25, and clathrin light chain B. The downregulation of MAP2 and SNAP-25 denoted lithium's possibility in normalising aberrant synaptic vesicle cycling and cytoskeletal instability that are unique to BD pathology. These proteins are essential to dock the synaptic vesicles and neurotransmitters upon their release and help lithium to recorrect synaptic dysfunction when administrated. Moreover, lithium facilitate effective control over oxidative stress and increase mitochondrial performance. Furthermore, lithium enhances reduced lipid peroxidation, improve mitochondrial complex I activity, and accelerate antioxidant gene expression during treatment. These results support the theory that mitochondrial dysfunction fuels BD pathophysiology and lithium's protective function stabilizes mitochondrial homeostasis by its neuroticizing action. Perhaps, calcium signaling is also considered essential since lithium downregulates transient receptor channels that interfere with endoplasmic reticulum (ER) stress pathways that regulate via changes associated with intracellular Ca2⁺ levels. These steps help to lower ER stress-induced death and preserve cellular integrity under oxidative or excitotoxic conditions brought by the BD. Furthermore, supporting lithium's ability to restore immune balance in BD patients could be achieved by suppressing proinflammatory cytokines such as TNF-α, & IL-6 and by enhancing anti-inflammatory mediators. The bidirectional link between inflammation and neuropsychiatric diseases suggest that the immunomodulation properties of lithium could greatly help to reduce BD symptoms. Importantly, the effect of lithium to phosphorylate CRMP2 and control microtubule dynamics exposes new cytoskeletal targets that might mediate both side effects and therapeutic action in BD. It is noteworthy that mood dysregulation and neurodevelopmental abnormalities have been linked to changes in CRMP2 activity, implying that lithium's stabilization of dendritic architecture may be fundamental in nature. In particular, the actions have a wide range to establish, lithium treatments have some restrictions also; to those who pretain thyroid and renal care, adverse effects must be under close observation. Differential responsiveness among BD subtypes emphasizes the need of precision medicine approaches, guided via biomarkers such GSK-3β phosphorylation levels or to their gene expression profiles. Ultimately, lithium's ability to modulate wide range of molecular targets that includes enzymes, cytoskeletal proteins, mitochondrial pathways, and immune mediators helps to explain its neuroprotective and mood-stabilizing actions. These multimodal activities place lithium as a special agent in the management of BD. Future studies will define the unique responses of lithium-based combination treatments and their technological advancements to greatly reduce the side effects of BD patients.

### 3.1 Limitation and future direction

Our findings however though exist quite preliminary in nature, the data retrieved relevant to cytoskeletal alteration linked MAP2 in mood disorders are insufficient for a secondary analysis. However, the strength of the present study underlies first-line exploration of microtubular disintegration associated with neural dysregulation and lithium dependent therapeutic approaches to neuroprotective effects. The study explained more feasible strategies to assess MAP2 levels of mood disorders via blood and CNS samples. Alongside, the weakness of the study encompass elimination of irrelevant publications that failed to bridge microtubule effect on mood disorders. Despite this, expanding large scale patient groups and clinical research analysis are further needed to evaluate better resolution of lithium as an effective drug in BD treatment. In addition, implimentation of other potential drugs as combination medicine to lithium, or its derivatives, neurosteroids, plant-derived compounds as effective antidepressents have to be investigated and explored. Furthermore, elucidation of specific biomarkers to diagnose neurodegenerative effects in earlier stages and their progressive reduction after treatment should be identified. Synthesis of univariate ideas for personalized medicines and amendments of new medical practices has to be clinically enforced. Moreover, advanced treatment possibilities and disease prediction aids via artificial intelligence (AI) and nanorobotics render paramount advantages over current medical practices to improve patient survival rates and disease eradication.

## 4 Conclusion

Since a period of half century, lithium is known as an effective maintainance drug to treat bipolar disorder. Several findings via meta-analysis, systematic studies and current observations indicated that BD has a life equivalency to that of the healthy population and Schizophrenia patients. Potetnial evidences from brain imaging studies after lithium admistration exhibited increased brain volume and enhanced grey matter density. However, in clinical settings, lithium was found to penetrate cellular boundaries due to its unique properties and show neurotrophic effects by regulating metabolic stimulators with undesirable side-effect profile. Although not much explained in the current review, the responsiveness of lithium to gene expression and protein regulation gives additonal features to futurestic therapeutic margins. Despite this, the introduction of new drugs in the recent decades reduced the prescribtion of lithium in developed countries. Although long time use of lithium shows varying health issues in BD patients, this effect was statistically not proven. Indeed, it is particularly important to consider that higher doses of lithium can be lethal to human in therapeutic relevance. Discovering well known side-effects, optimised kinetics, and regular monitoring of serum-lithium levels can keep treatment safety to BD patients. Deep studies of lithium with other drugs as a combination therapy will gain intense therapeutic possibilities against BDs and other neurological diseases. Seeking ultimate knowledge and better clinically relevant pathophysiological targets based drugs will ultimately improve the quality of life and survival rates for those who suffer from devasting disorders.

## Funding

We are thankful for the support provided by UCSI University for the support provided by the Center of Excellence for Research, Value Innovation and Entrepreneurship (CERVIE) and Research Excellence and Innovation Grant (REIG) with code REIG-FPS-2025/038.

## CRediT authorship contribution statement

**MT:** Writing - review & editing, Formal analysis. **SR & SC:** Writing - original draft, Supervision, Visualization, Writing - review & editing, Formal analysis. **MA & NA:** Software, Writing - review & editing, Formal analysis. **EK:** Writing - review & editing, Formal analysis. **PS:** Writing - review & editing, Formal analysis. **CP:** Writing - review & editing, Formal analysis. **RM**: Conceptualization, Supervision, Methodology, Software, Formal analysis, Funding acquisition, Data curation.

## Declaration of Competing Interest

The authors declare no conflict of interest.
